# Visceral Leishmaniasis in Bolivia: Current Status

**DOI:** 10.1590/0037-8682-0421-2019

**Published:** 2020-10-21

**Authors:** Juan Sergio Mollinedo, Zoraida Aymara Mollinedo, Wilson Julio Gironda, René Edmundo Mollinedo, Pavel Mollinedo, Oscar D. Salomón

**Affiliations:** 1 Instituto de Salud y Medio Ambiente, La Paz, Bolivia.; 2 Universidad Amazónica de Pando, Pando, Bolivia.; 3 Sociedad Boliviana de Entomología, La Paz, Bolivia.; 4 Ministerio de Salud, La Paz, Bolivia.; 5 Instituto Nacional de Medicina Tropical, Puerto Iguazú, Misiones, Argentina.

**Keywords:** Visceral leishmaniasis, Leishmania infantum, Lutzomyia longipalpis, Bolivia

## Abstract

**INTRODUCTION:**

In Bolivia, before 1982 there were no records of visceral leishmaniasis (VL) cases that would allow us to review and describe the temporospatial occurrence of VL by ecoregions in provinces and departments of Bolivia to evaluate its impact on public health, risk of outbreaks, or dispersion.

**METHODS:**

This update on VL in Bolivia is based on research, reviews, and retrospective literature analyses of online data and libraries and institutional reports, from 1939 to the present.

**RESULTS:**

In Bolivia, 56 cases of VL have been reported. Until 2014, only three endemic departments had been identified (La Paz, Santa Cruz, and Tarija). Since then, further cases have been recorded in Pando, Cochabamba, and Beni, and in Chuquisaca in 2015. In Yungas, a VL focus was confirmed by isolating and comparing parasites from human and dog cases, and from the *Lu. longipalpis* vector. VL cases from seven departments, involving 12 different ecoregions were located within the Amazon and Plata basins.

**CONCLUSIONS:**

We confirmed that dogs are its primary reservoir, and *Lutzomyia longipalpis* is its main vector (currently dispersed in six departments). The primary vectors in areas where *Lutzomyia longipalpis* is absent are *Migonemyia migonei* and *Lutzomyia cruzi*.

## INTRODUCTION

Official attention to neglected tropical diseases (NTD) in Bolivia, including leishmaniasis, has been limited in focus due to the misconception of "highland country" (Andean country); Authorities, Visceral leishmaniasis (VL) researchers, and health care professionals need to better understand the vulnerability of the native population, which inhabits an endemic tropical area that covers 60% of the nation’s territory. Our purpose is to contribute evidence to broaden public awareness of the population’s exposure to Visceral leishmaniasis in Bolivia^1^.

## METHODS

### Study Areas

Bolivia is located at 10° to 23° south latitude. It is divided into nine departments covering a surface area of 1,098,581 km². Bolivia is also divided into three physiographic regions: an Andean Zone, a subtropical Zone, and a tropical Zone. VL (Visceral Leishmaniasis) is transmitted along the river basins and certain ecoregions of the latter two zones: the Subtropical zone in the center-south of the State covers 13% of the territory (142,815 km²), with altitude decreasing from 2,500 to 900 m (from west to east), mild to warm climate, from 15 to 25°C. The tropical Zone in the north, northeast, east, southeast and south of Bolivia covers 59% of the national surface (648,163 km²). It is a vast plain with low plateaus covered with widespread forests at altitudes of 900 m and below. The zone has an average annual temperature of 22 to 25°C. Both zones house two river basins, the Amazon, which covers 65.9% of the country, and the Plata, which covers 20.9%.

### Vector status

In Bolivia, phlebotomine (sandflies) are found in a wide array of habitats spanning altitudes of 170 to 2700 m^2^ ^,^ ^3^. In general, insect captures were carried out using a) protected human bait, b) CDC light traps, and c) an illuminated Shannon trap, which were installed in jungle, peri-urban, and urban stations, during maximum insect activity periods, or during the night. The insects were taxonomically identified by genitalia. The females were dissected, and parasites were isolated from infected digestive tracts by culture and/or amplification in hamsters, then preserved in liquid-nitrogen for later molecular studies. *Lu. longipalpis* (the main VL vector) inquiries were carried out in the departments of La Paz, Beni, Santa Cruz, Pando, and Tarija during the periods described above.

### Reservoirs

In the literature, various species of medium and small wild mammals (Order Marsupialia, Xenarthra, Rodentia, Carnivora, Primata, Chiroptera) have been found infected with *Leishmania* sp. parasites. In Yungas, there are no wild dogs, or they are extremely rare within the focus. Therefore, various attempts have been made in La Paz, Beni and Tarija departments to define the main VL reservoirs. Captured wild mammals, and clinically suspicious dogs were studied by autopsy. Samples of liver, spleen, and bone marrow were taken, and used to seed culture media and inoculate hamsters.

### State of the art leishmaniasis response methods in Bolivia

### Diagnosis

VL diagnosis is the Programa Nacional de Control de la Leishmaniasis’s (PNCL’s)^4^main difficulty due to multiple factors. First, rural areas are remote and difficult for health services to access^5^ ^-^ ^6^. Second, the disease clinical manifestations are not very specific^7^. Third, the VL-specific experience of doctors from rural areas is limited^8^ ^,^ ^9^ ^,^ ^10^. Fourth, the region’s laboratory network does not support referring patients to a more specialized, better equipped level for appropriate diagnostic sampling. Finally, the lack of minimal health center equipment for performing iliac crest puncture, or other types of sample aspiration necessitates patient referral to specialized city laboratories causing families to incur higher expenses. The PNCL has not yet established "rapid diagnostic tests" such as the Direct Agglutination Test (DAT), rk39-ICT, serological tests (IFAT, ELISA), or the PCR technique used only in specialized research centers.

### Treatment

The PNCL-recommended therapy used in Children's Hospital is Meglumine Antimoniate (Glucantime®, Sanofi Aventis, Paris, France) with intravenous or intramuscular injections at a dose of 20 mg SbV/kg/day for 30 days, which generally yields good results^4^.

### Vectors

To date, 121 species of Phlebotominae have been recorded in Bolivia^11^. Phlebotomine distribution maps are incomplete, both at local and national levels. The main VL vector is *Lutzomyia longipalpis*, which is a complex species whose intra-specific taxonomic level is under discussion^12^. Other sandfly species close to *Lu. longipalpis* include *Lu. cruzi* ^13^ ^,^ ^14^ and less related species that have adapted to environments with anthropic modifications, such as *Migonemyia migonei* ^15^ ^,^ ^16^, can act as *L. infantum* vectors in VL areas where *Lu. longipalpis* is not present, or in scenarios in which two or more species display sympatric presence.

*Lutzomyia longipalpis* (Lutz and Neiva, 1912) acts as a primary vector. In 1973, Jorge Velasco reported that 78% of catches in Yungas were *Lu. longipalpis* specimens. Le Pont F. captured *Lu. longipalpis* in forest and domestic, but mainly in peri-domestic zones in Yungas, finding it to be the most competent anthropophilic vector among 14 species studied in or near dwellings^2^. This species displayed relative abundance peaks during dry seasons, and constituted 98% of peridomicile protected human bait catches, where it also showed an attraction to chickens, dogs, and pigs. *Leishmania* sp. infection rates determined by dissection and direct observation were between 0.8 and 4.2%, according to capture site, incriminating *Lutzomyia longipalpis* as Yungas’ VL vector^17^.

Prior to 2017, four distribution regions in Bolivia, and two *Lu. longipalpis* morpho-types had been described: The 1S phenotype in the aforementioned focus in La Paz, and the 2S phenotype found in Santa Cruz and in two Andean areas between 1,800 and 2,700 m in the towns of Chuquisaca and Potosí^18^ ^-^ ^20^. In 2017, *Lu. longipalpis* presence was recorded in Assis town, Acre state, tri-national border Brazil, Bolivia, and Peru^21^. In 2018, specimens were found in Yacuiba and Tarija (Magne M., pers.com). These last two descriptions are accompanied by human VL cases, which makes us assume *Lu. longipalpis* phenotype 1S dispersion in these two regions (Pando and Tarija), necessitating further studies.

*Lutzomyia cruzi* (Mangabeira 1938). In 2010, in El Carmen town, Busch province, *Lu cruzi* presence was described^11^. Le Pont F. (pers. Com.) in 2004 also recorded *Lu. cruzi* as the most abundant species in the same department (Robore, Aguas Calientes, Santiago de Chiquitos) and in small towns near the Pilcomayo River (Puerto Margarita, kapactala and Saapuco) in Tarija. Both its presence, and its current or potential importance as a VL vector should be defined in the Bolivian Pantanal of the department of Santa Cruz’s border with Brazil^14^.

*Migonemyia migonei*. This species was recently associated with natural *L. infantum* infection in the VL endemic zone of Pernambuco, Brazil^15^. It has also been implicated as a VL vector in Argentina^16^. Its presence in Bolivia has been described in Yungas, in the forest canopy of the Alto Beni region, and with greater abundance in the Chaco lowlands that drain into the Río de la Plata basin^2^. However, the species’ capacity to serve as a vector among local populations remains to be verified before it can be implicated as a *Lu. Longipalpis* vector.

Other species. Phlebotominae species have been described as potential vectors based on their presence in disease foci, their high anthropophilicity, and identification of *Leishmania infantum* DNA in the insect digestive tract. *L. infantum* DNA sequences have been obtained by PCR-RFLP in *Ny. neivai* and *Ny. whitmani* ^22^. Both insect species are registered in Bolivia, and thus deserve further research^2^.

### Reservoirs

Our investigations of wild animal disease reservoirs did not reach a conclusive result. We only isolated one enzymatic *Leishmania hertigi* variant^23^.

In 2001, in the department of Tarija, 148 specimens of 9 wild mammal species were captured that could not be processed to detect the disease, because of a health quarantine declaration due to the presence of Hanta virus presence in the area during that period^5^.

Regarding domestic reservoirs, in 1982, *L. infantum* was isolated from five dogs (*Canis lupus familiaris*) from the Yungas area with serological confirmation^24^. Similarity was seen in 13 enzymes between isolates from human strains (clinical disease), sick dogs (domestic reservoir), and *Lu. longipalpis* (vector). In Yungas, most dogs with the disease die around two to three years-old. Dogs five or more years-old are infrequently found^25^.

### Control of sandflies:

Indoor use of insecticides widely used for 62 years (1956-2018) for "malaria control" displays a marked chronology of the introduction and retirement of different chemical agents: Organochlorines 1956-1993, Pyrethroids 1993-2013; Carbamates 2014-2018. These treatments, coupled with other vector control initiatives decreased endemic malaria municipalities from 148 to 19 by 2018. Many of the 129 municipalities, where insecticide is no longer being fumigated on walls, was endemic to malaria and leishmaniasis. Thus, a potential collateral benefit to reducing abundance of vectors other than anophelines has been eliminated. 

Le Pont in 1989 conducted a residual spray study with deltamethrin (0.025 g/m^2^) in Sud Yungas assessing the impact of the intervention in phlebotome. He recorded a pretreatment average of 16.7 sandflies/night/CDC light trap, and managed to eliminate *Lutzomyia longipalpis* from houses and animal shelters by 9 and 10 months post-treatment, respectively^26^.

### Reports analysis

This review is made based on national institutional reports, medical records from Children's Hospital, and the results of 36 years of work and research at the UPAMET (Unidad de Parasitología y Medicina Tropical) of IBBA (Instituto Boliviano de Biología de la Altura), the LNPE-INLASA (Laboratorio Nacional de Parasitología y Entomología of the Instituto Nacional de Laboratorios de Salud), the PNCL (Programa Nacional de Control de la Leishmaniasis), health related documentation, and previous publications. This search allowed us to find and review all recorded VL cases up to 2010, the year in which notifications began to be registered by SNIS-VE (Servicio Nacional de Información en Salud - Vigilancia Epidemiológica).

## RESULTS

**FIGURE 1:**
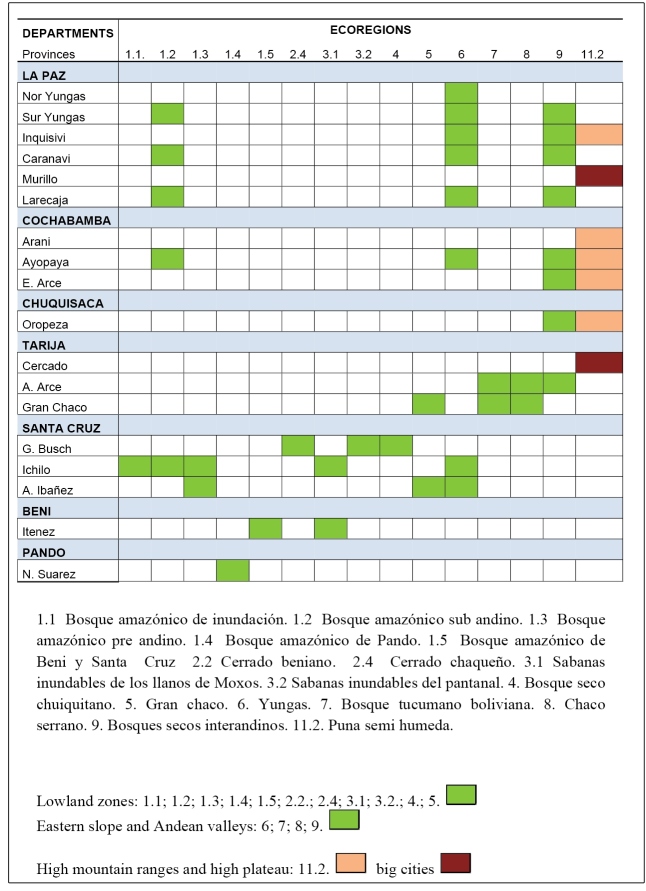
Seven departments covering 18 provinces (12 Ecoregions with autochthonous VL reported cases); Bolivia. 2019.

Ecoregions dissemination (Figure 1):

Bolivian internal human migration from highlands to lowlands (colonization) beginning in the 1980s and linked to socio-economically motivated development projects, caused important anthropic impacts on the environment. This activity continues the degradation and invasion of natural ecosystems, leading to significant change. One unforeseen consequence was an increase in CL incidence at the national level, with more than 3,000 cases/year and a trend toward VL dispersion^4^ ^,^ ^5^. Both phenomena were linked to increasing migrant arrivals into endemic areas, and environmental changes including uncontrolled deforestation and unplanned urbanization, generating outbreaks with peri-urban transmission cycles for both clinical forms of leishmaniasis in different small and intermediate cities in the departments of La Paz, Tarija, and Pando (unpublished data).

To better understand these trends, CL and VL investigations were carried out in different ecoregions, in four of the seven departments, using native VL case reports.

The area of La Paz has three ecoregions (Yungas, bosque amazónico sub andino, and bosques secos interandinos). 

Santa Cruz has two zones, an old endemic zone in Germán Busch province, and the southeast of Bolivia (Mato Grosso do Sul state border, Brazil), formed by three ecoregions. In two of these (cerrado chaqueño and bosque seco Chiquitano) seven cases have been reported. 

One study focused on the two provinces in the department of Tarija: Arce and Gran Chaco, to the south of Bolivia in Salta province border, Argentina, where six cases have been reported within the potential transmission area. Furthermore, the SNIS-VE has reported VL cases in some provinces of the departments of Cochabamba, Beni, and Pando (2014), and in Chuquisaca (2015).

### Human cases

### Pioneering research (1939 - 1981)

The first three historical reports are: Gatti et. al. (1939); Barros and Rosenfeld (1942); and Arruda et. al.(1949); these provided no certain tracing of the origins of their infections, but we add them to the record of general VL cases in Bolivia.

### Human cases from 1982 - 2018

Seventy nine VL cases have been reported spanning 36 years (1982 - 2018). Fifty six of these had Visceral Leishmania, and 23 had *Leishmania* sp. Infections. Most cases were in malnourished children from rural areas found in 18 provinces (15 ecoregions where the transmission cycle can be developed) in seven departments. Patients with VL have been reported in areas endemic for cutaneous leishmaniasis, mainly with *L. (V.) braziliensis*. Families that had some knowledge of cutaneous leishmaniasis and the economic resources to transfer their patients to a main city, did so, so that they could be diagnosed and treated in reference centers. Table 1.

**TABLE 1:** Distribution of human VL cases in Bolivia by department, province, and year reported (1939 to 2018).

Department	Time Periods				Total
Province	prior 1982	1983-2001	2002-2011	2012-2018	
**La Paz**	**3**	**8**	**4**	**10**	**25**
Nor Yungas	1	3		4	
Sur Yungas	2	3	1	2	
Inquisivi			3	1	
Caranavi		2			
(*) Murillo				2	
Larecaja				1	
**Santa Cruz**	2	1	3	3	9
German Busch	2	1	3	1	
Ichilo				1	
A. Ibañez				1	
**Tarija**				6	6
(*) Cercado				4	
Arce				1	
Gran Chaco				1	
**Pando**				1	1
Nicolas Suarez				1	
**Cochabamba**				**13**	**13**
(**) Arani				11	
Ayopaya				1	
E. Arce				1	
**Beni**				1	1
Itenez				1	
**Chuquisaca**				1	1
(**) Oropeza				1	
**Total Bolivia**	5	9	7	**35**	**56**

(*) Hospitals in big cities that are not in an endemic zone. (**) Provinces with ecoregions in which phlebotomines are not present.

**FIGURE 2:**
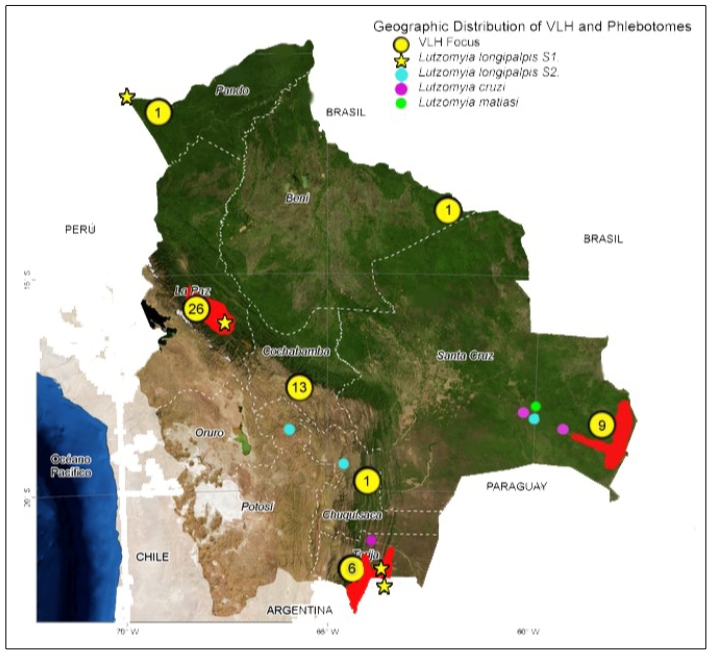
Map of Bolivia with number of notifications of human VL cases, endemic foci, and distribution of *Lutzomyia longipalpis* (phenotypes S1 and S2), *Lu. cruzi* and *Lu. matiasi*; La Paz department is the focal site where 26 human cases dispersed across five provinces were registered: Nor Yungas, Sud Yungas, and Caranavi, without data from Inquisivi and Larecaja provinces. Santa Cruz department as second Zone with 9 registered human cases: Ichilo A. Ibáñez and G. Busch provinces. Tarija department is the third Zone, with 6 human cases registered in A. Arce province. Map source: JPL Global Imagery Service: http://wms.jpl.nasa.gov/wms.cgi. Our elaboration, 2019.

Our investigations (Figure 2 and Table 2).

**TABLE 2:** Human cases of Visceral leishmaniasis in Bolivia (1939-2018) , clinical and epidemiological information.

Clinical/epidemiological	Periods for years			
information	prior to 1982	1983-2001	2002-2011	2012-2018
**SEX**	**5**	**9**	**7**	35 (*)
Male	4	8	6	24
Female	1	1	1	15
No data	0	0	0	0
**AGE**				
Children (< 5 years)	2	7	6	3
Teenagers (6 - 17 years)	0	1	1	1
Adults > 18 tears)	3	1	0	?
No data	0	0	0	31
**FEVER**				
Present	5	9	7	4
No data	0	0	0	31
**HEPATOSPLENOMEGALY**				
Present	4	8	7	4
No data	1	0	0	31
**ADENOPATHY**				
Present	2	9	5	4
No data	3	0	2	31
**PANCYTOPENIA**				
Present	1	8	3	3
No data	4	1	4	32
**DIAGNOSTIC**				
Clinical	5	1	7	35
Laboratory (smear)	4	8	7	4
quick tests	0	0	0	?
others (culture-PCR)	0	1	2	0
**TREATMENT**				
pentavalent antimonial	3	9	7	4
others	0	0	0	?
No data	0	0	0	31
**EVOLUTION**				
Healing	3	7	7	4
Death	2	2	0	?
No data	0	0	0	31

(*) For the last 14 years, the ministry of health has provided limited global data. Medical records remain at the first level.

**FIGURE 3:**
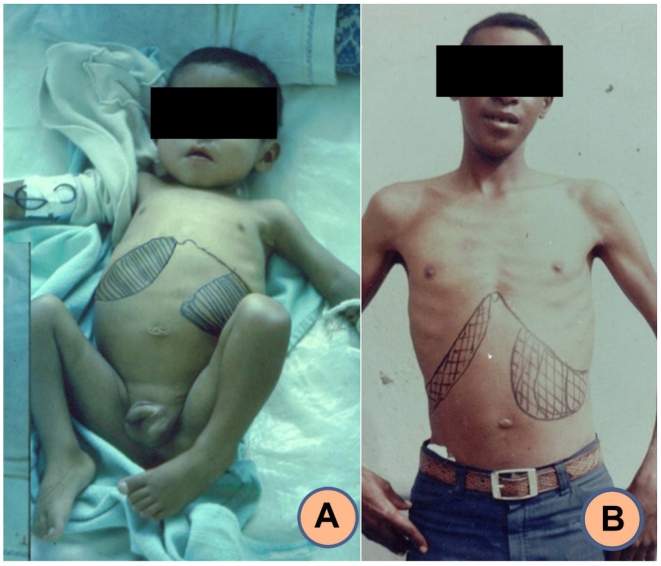
**Photo A:** Two year-old male child from South Yungas; La Paz department. Mollinedo S. (1982). **Photo B:** Sixteen year-old male from North Yungas; La Paz Department. Urgel & Desjeux (1987). Both with classic clinical fever, splenomegaly, hepatomegaly, and poliadenopathy.

In 1982 at La Paz city Children's Hospital, we found a boy who was two years and two months old, from Sud Yungas, with classic signs/symptomology, from whom *Leishmania infantum* was isolated and typed. He was recognized as the "First indigenous case of Visceral Leishmaniasis in Bolivia". (Figure 3-A)^1^. His treatment was guided by reference to a previous finding in a similar clinical case that occurred in the same Hospital, in a two-year-old girl from North Yungas. Autopsy slides confirmed presence of amastigotes in her spleen and liver (M S., unpublished data). In later years, during a field investigation in North Yungas (the area the first two patients came from), a pair of infected sibling children were found based on classic signs/symptomology (1987); (Figure 3-B; cited in Urjel & Desjeux^2^). One sibling was 5 years old and the other was 16. In 1993, we tended a female patient from Caranavi, with clinical characteristics corresponding to Diffuse Cutaneous Leishmaniasis (DCL) and abdominal swelling. Two etiological agents, *L. infantum* and *L. amazonensis*, were identified by isolation and isoenzymatic typing^7^. In 2002, a patient from Taipiplaya town admitted to Children's Hospital in 1999 became the 14^th^ recorded case of VL, leading us to undertake a cross-sectional survey of VL in that locality by serological and molecular tests, involving 122 clinically healthy individuals. A whole community meeting was convened to obtain informed consent, after previous meetings with local authorities. Fourteen point four percent of the examined population had anti-rk39 antibodies, confirming the spread of *Leishmania infantum* among residents of that area^9^. An outbreak investigation we carried out in the department of Tarija (1997 to 2002), considered at that time a new area of endemicity for cutanous leishmaniasis^5^, allowed us to identify children clinically suspected of VL. Therefore, in 2003 seven communities in Arce province were selected for a clinical and entomological study. With prior informed consent and permission from local authorities, 732 samples were taken, among which five cases (0.7%) were positive in an ELISA-rk39 test^10^. Table 1.

Human cases from 2010 to 2018 (Figure 2 and Table 1).

Thirty-five partially confirmed cases were reported by the SNIS-VE between 2010 and 2018. In 2014 for the first time, three departments reported presence of VL cases: Pando (one case), Cochabamba (eleven cases), and Beni (one case). In 2015, one case came under study by the ministry of health in Chuquisaca. A Medical Records review of La Paz city Children's Hospital covering the last ten years (2009 to 2018) idetified four additional diagnosed VL cases thath were not included in the SNIS-VE records. Table 1. 

Based on SNIS-VE notifications, we consider Arani provinces in Cochabamba, and Oropeza provinces in Chuquisaca to have extremely low to no likelihood of vectors. This conclusion is supported by notification records from large city hospitals located in the Andean zone where there is no transmission (absence of disease, reservoirs, and vectors).

## DISCUSSION

One hundred and eighteen years after the discovery of the first VL case in South America, this disease is currently one of the most neglected, and mainly affects disadvantaged human populations, such as those living in developing countries with poor sanitary conditions, and populations involved in anthropic environmental transformations in subtropical and tropical areas. Both of the above characteristics frequently manifest in Bolivia. Therefore the main objective of the present study is to contribute compiled evidence from 19 national references, five national meetings of leishmaniasis officials/representatives with local and regional representatives, and technical reports, to broadly disseminate knowledge of the state of VL transmission throughout the Bolivian Health System.

In Bolivia, there is a significant presence of dogs infected with *L. infantum* ^24^, high densities of the main vector *Lu. longipalpis* ^2^ ^,^ ^17^ and other potential vectors (*Lu. cruzi* and *Mg. migonei*), in addition to a number of homogeneous ecological areas shared with neighboring countries with well known established VL foci, including Mato Grosso do Sul, Brazil^27^ ^,^ ^28^ and Salta, Argentina^16^ ^,^ ^22^ ^,^ ^29^. The VL epidemiology centering in ​​Bolivia - Mato Grosso do Sul border area should be similar on both sides of the border. However, the number of VL cases reported in Bolivia is minimal, apparently due to underreporting; in LC it is over 72% in some regions^30^. This discepancy may be explained by several factors: health professionals unaccustomed to this disease, lack of access to health services for Bolivian residents in a territory undergoing continuous expansion of its agricultural frontier, a national public health system that lacks a diagnostic routine coupled with a laboratory network lacking VL diagnostic tests, changes in human behavior associated with massive migration, and cross-border diagnoses of indigenous cases not registered to their place of origin.

We have identified three outbreaks of VL prior to 2009, and between 2014-2015 national health authorities have reported four other outbreaks (discovered due to a study by the ministry of health). These regional health events happen in the context of vector urbanization and dispersion of VL parasite transmission mechanisms, which began in the 1970s in northeastern Brazil. Between 2000-2013 it was already generating outbreaks in southern Brazil, northern Argentina, Paraguay, and Uruguay. At the same time, along Bolivian borders with these countries, transmission and presence of vectors was being reported. *Lu. longipalpis* genetic structure along these borders suggested possible hybridization of a recently arisen urban dispersive population with ancestral rural populations, thus permitting a disease with rural endemic and scattered transmission to become epidemic and urban. 

Current *Lu. longipalpis* records in six departments, four of these with human VL cases (La Paz, Santa Cruz, Pando and Tarija), show a new VL epidemiological scenario in Bolivia.

The recently created Unified Health System (SUS - Sistema Único de Salud) in Bolivia (2019) faces as its main challenge the sustained implementation of surveys and laboratory tests over medium and long terms, being necessary to strengthen health promotion and risk prevention efforts among populations living in endemic areas, improving health population coverage (My Health Program), strengthening the quality of diagnosis at all levels, and treatment and epidemiological surveillance.

Considering the experience obtained during the last years building "regional reference centers", the human resources training needed to form multidisciplinary teams that can develop operational research programs funded by sustainable financing sources is a requisite to confirm new outbreak notifications, reduce relevant knowledge gaps, and assure timely provision of diagnostic supplies (mainly VL diagnosis), medications, and appropriate case management.
